# Post-genomics of the model haloarchaeon *Halobacterium *sp. NRC-1

**DOI:** 10.1186/1746-1448-2-3

**Published:** 2006-03-16

**Authors:** Shiladitya DasSarma, Brian R Berquist, James A Coker, Priya DasSarma, Jochen A Müller

**Affiliations:** 1 University of Maryland Biotechnology Institute, Center of Marine Biotechnology, 701 E. Pratt Street, Suite 236, Baltimore, MD 21202, USA; 2 Department of Biology, Morgan State University, 1700 East Cold Spring Lane, Baltimore, MD 21251, USA

## Abstract

*Halobacterium*****sp. NRC-1 is an extremely halophilic archaeon that is easily cultured and genetically tractable. Since its genome sequence was completed in 2000, a combination of genetic, transcriptomic, proteomic, and bioinformatic approaches have provided insights into both its extremophilic lifestyle as well as fundamental cellular processes common to all life forms. Here, we review post-genomic research on this archaeon, including investigations of DNA replication and repair systems, phototrophic, anaerobic, and other physiological capabilities, acidity of the proteome for function at high salinity, and role of lateral gene transfer in its evolution**.**

## Background

Halophilic archaea (haloarchaea) are extremophiles that grow optimally under conditions of extremely high salinity, 5–10 times that of seawater [1]. They contain a similarly high concentration of salts internally and exhibit a variety of novel molecular characteristics, including acidic proteins that resist the denaturing effects of salts, and DNA repair systems that minimize the deleterious effects of desiccation and intense solar radiation. In addition, haloarchaea are metabolically versatile, exhibiting phototrophic and facultative anaerobic capabilities. Significantly, their ease of culturing and genetic tractability have made them model experimental organisms and facilitated the use of isogenic strains for rigorous post-genomic studies.

Classical studies of haloarchaea contributed significantly to our understanding of adaptive mechanisms, as well as universal features of life [2]. Notable discoveries originally made using haloarchaea include the S-layer glycoprotein cell wall [3], branched-chain ether lipids [4] and light-driven proton pump, bacteriorhodopsin [5], in the cell membrane, and metabolic and biosynthetic processes operating intracellularly at saturating salinity [6]. Demonstration of light-driven ATP synthesis by reconstituted lipid vesicles containing bacteriorhodopsin and mitochondrial ATPase provided proof of Mitchell's chemiosmotic coupling hypothesis [7]. These early discoveries established the value of studying diverse microbes from the environment and set the stage for phylogenetic studies leading to the three-domain view of life [8].

In 2000, the complete genome sequence of *Halobacterium *sp. NRC-1 [9-11], a typical haloarchaeon widely distributed in hypersaline environments, such as solar salterns and the Great Salt Lake, Utah, USA [12], became available. More recently, four additional haloarchaeal genomes have been or are currently being sequenced: *Haloarcula marismortui*, a metabolically versatile microorganism from the Dead Sea [13], *Haloferax volcanii*, a prototrophic and moderate halophilic microorganism from Dead Sea mud [14], *Natronomonas pharaonis*, an alkaliphile from the soda lakes of the Sinai [15], and *Halorubrum lacusprofundi*, a cold-adapted extreme halophile from an Antarctic lake [16]. These five genomes provide an excellent view of haloarchaeal diversity. Here, we review what has been learned about the first sequenced haloarchaeon, *Halobacterium *sp. NRC-1, primarily through post-genomic studies [11].

## *Halobacterium *sp. NRC-1

**Figure 1:**
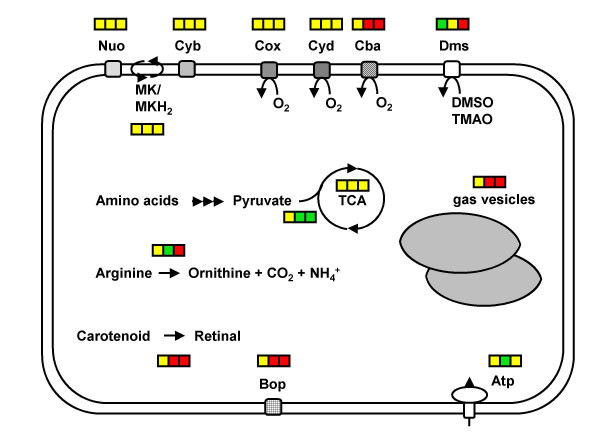
**Physiology and transcriptomics of *Halobacterium *sp. NRC-1. **An overview of relative transcript levels of selected genes is indicated, along with metabolic characteristics of the cell, indicated by rectangular box. The double line depicts the cell membrane. Square boxes, left to right: transcription levels in cells grown by aerobic respiration, anaerobic respiration using TMAO, and fermentation. Green, yellow, and red boxes indicate reduced, unchanged, and increased transcript levels, respectively. Abbreviations: Nuo: NADH oxidoreductase; MK/MKH_2_: oxidized/reduced menaquinone; Cyb: cytochrome *b_6 _*oxidoreductase; Cox: *aa*_3_-type cytochrome oxidase; Cyd: quinol *bd *oxidase; Cba: *ba*_3_-type oxidase; Dms: DMSO/TMAO reductase; TCA: tricarboxylic acid cycle; Bop: bacterio-opsin; Atp: ATP synthase

*Halobacterium *sp. NRC-1 is an extreme halophile (with a 4.3 M NaCl optimum) that grows best heterotrophically in a rich organic broth. However, the organism is metabolically versatile (Fig. 1); in addition to its aerobic metabolic capacity, it possesses facultative growth capabilities through anaerobic respiration, utilizing dimethyl sulfoxide (DMSO) and trimethylamine *N*-oxide (TMAO), and via arginine fermentation. It also has phototrophic capability through the light-driven proton pumping activity of the retinal protein, bacteriorhodopsin, which is organized into a two-dimensional crystalline array in its purple membrane. *Halobacterium *sp. NRC-1 cells are highly motile, synthesizing gas vesicles, which are hollow protein structures, intracellularly for buoyancy and flotation, and sensory rhodopsins for phototaxis. Relevant to the study of genetic regulation, *Halobacterium *sp. NRC-1 responds to many environmental effectors, including high and low temperatures and salinities, and ultraviolet (UV) and ionizing radiation.

A major advantage of studying *Halobacterium *sp. NRC-1 is its ease of manipulation in the laboratory. Culturing is simple, with a 6 hour generation time at 42°C [17]. Additionally, it is genetically tractable, being transformable at high-efficiency [18], and a good selection of cloning and expression vectors are available [19]. Several genetic markers have been developed, including selectable markers for mevinolin resistance, as well as the selectable and counterselectable *ura*3 gene, which permit construction of systematic gene knockouts and replacements [20-22]. Whole-genome DNA microarrays have been used successfully to interrogate patterns of gene expression [23-26]. *Halobacterium *sp. NRC-1 cells are easily lysed in hypotonic medium, releasing both soluble and membrane proteins for biochemical and biophysical studies [17]. These characteristics, together with the availability of the complete genome sequence, have made *Halobacterium *sp. NRC-1 an excellent model microorganism for research as well as for teaching.

### Genome, genes, and proteins

*Halobacterium *NRC-1 possesses the smallest genome to date among halophiles. It is 2,571,010 bp in size, and is composed of a large GC-rich chromosome (2,014,239 bp, 68 % G+C), and two smaller extrachromosomal replicons, pNRC100 (191,346 bp) and pNRC200 (365,425 bp), with 58–59 % G+C composition [9-11]. The two smaller replicons contain 145,428 bp of identical DNA and 33–39 kb inverted repeats catalyzing inversion isomers [27], and the majority of the 91 IS elements, representing 12 families, found in the genome [10,11]. As a result of the large number of repeated sequences, genome assembly required extensive genomic mapping and an ordered clone library of pNRC100 [9,10][27]. Of the 2,630 likely protein-coding genes in the genome, 2,532 are unique. *Halobacterium *predicted proteins were found to be highly acidic [27] and a substantial number had bacterial homologs as their closest relatives, suggesting that they might have been acquired through lateral gene transfer [28]. In addition, 52 RNA genes were also identified; however, the 16S rRNA sequence and other unique characteristics did not allow placement within a validly described *Halobacterium *species, and this point has been the subject of some controversy [29-31]. Interestingly, about 40 genes in pNRC100 and pNRC200 code for functions likely to be essential or important for cell viability (e.g. thioredoxin and thioredoxin reductase, a cytochrome oxidase, a DNA polymerase, multiple TATA-binding proteins (TBP) and transcription factor B (TFB) transcription factors, and the only arginyl-tRNA synthetase in the genome). As a result, these replicons were suggested to be essential "minichromosomes" rather than megaplasmids [9]. Several reports on annotation of the *Halobacterium *sp. NRC-1 genome have been published [9-11].

## Development of experimental tools

A key factor in the development of *Halobacterium *sp. NRC-1 as a model system has been its genetic tractability [19]. Transformation was accomplished by employing EDTA to chelate Mg^2+^, thereby weakening the S-layer, and resulting in formation of spheroplasts, followed by treatment with polyethylene glycol, which induces competence [18]. Plasmid vectors were derived from large extrachromosomal replicons, e.g. pNRC100 [32], or natural miniplasmids, such as pHSB [33]. For example, a popular shuttle vector, pNG168 [34], contains the pNRC100 minimal replicon for replication and Mev^r ^marker for selection in *Halobacterium*. The Mev^r ^marker contains an up-promoter allele of the *Haloferax volcanii *3-hydroxy-3-methylglutaryl-CoA reductase (*mva*) gene, required for branched chain lipid biosynthesis [35].

**Figure 2:**
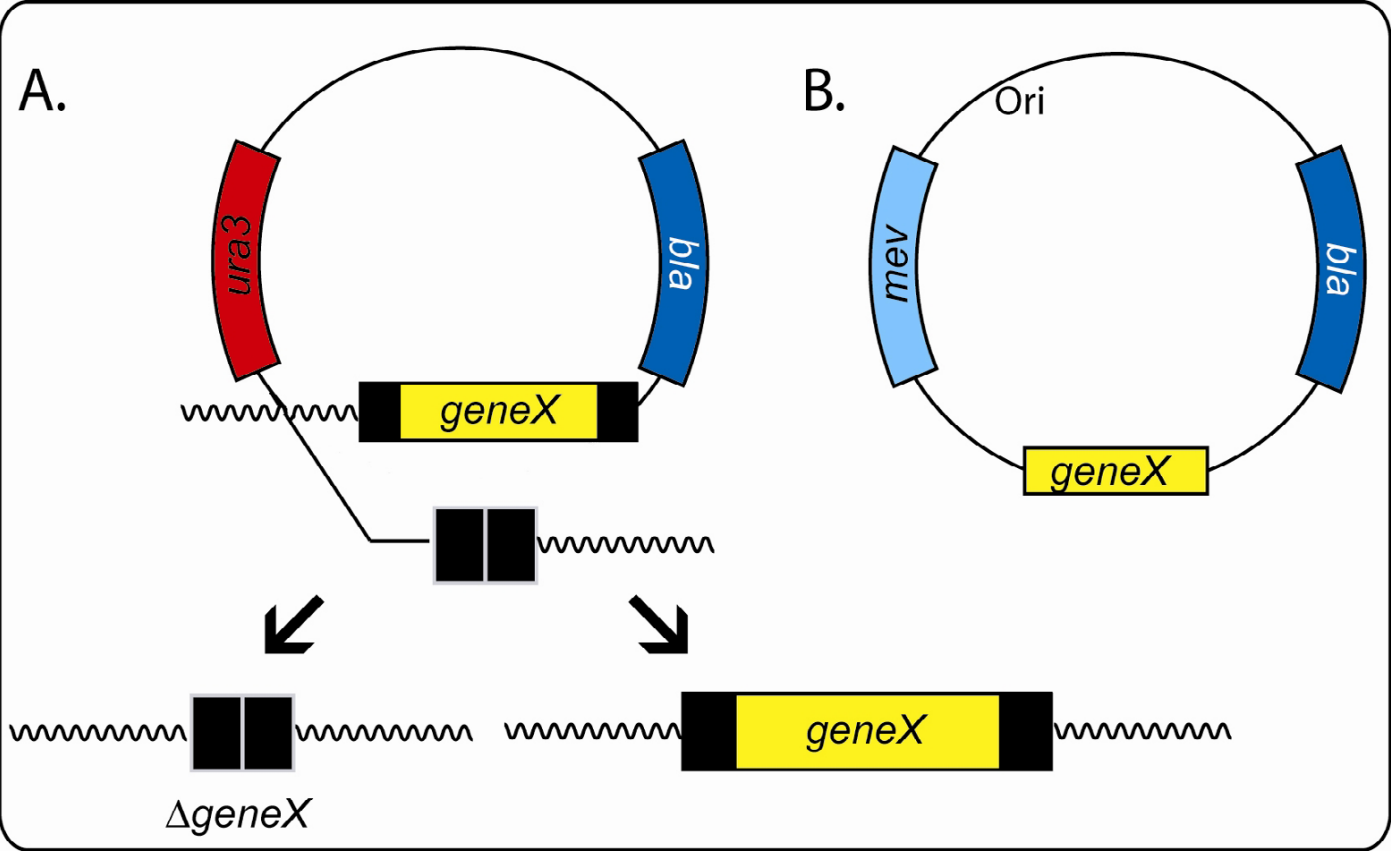
**Gene knockout strategies in *Halobacterium *sp. NRC-1. **A. *Nonessential genes*. After a suicide vector containing a deletion of a gene of interest (*gene*X) is integrated into the *Halobacterium *Δ*ura*3 host strain by selection of uracil prototrophy (Ura^+^), plasmid excision is selected with 5-fluoroorotic acid resistance (Foa^r^), resulting in either replacement with the deleted allele (Δ*gene*X) (left) or restoration of the wild-type allele (right). For genes that are essential for cell viability, only wild-type gene alleles are recovered. B. *Essential genes*. A pseudo-complementation strategy is used where an autonomously replicating plasmid vector which contains the functional gene of interest, *gene*X, is introduced into the host strain, e.g. by selection for mevinolin resistance (Mev^r^). Knockout of the chromosomal copy may then be selected using selections described in part A.

A directed gene replacement and knockout method for *Halobacterium *sp. NRC-1, the first described for an archaeon [20-22], exploits the selectable and counterselectable properties of the *ura*3 gene (Fig. 2). This gene codes for orotidine 5'-phosphate decarboxylase required for pyrimidine biosynthesis [36]. In this approach, a target gene allele (e.g. a deletion or point mutation) is first cloned into a suicide plasmid (e.g. pBB400) capable of replication in *E. coli *(but not in *Halobacterium*); the plasmid also contains the *ura*3 gene under the control of its own promoter. The resulting plasmid is introduced into a *Halobacterium *Δ*ura*3 host via transformation. Integrants are then selected by uracil prototrophy (Ura^+^) using commercially available uracil-dropout media components. Subsequently, plasmid excisants are selected via *ura*3 counterselection, 5-fluoroorortic acid-resistance (Foa^r^), giving rise to derivatives containing either the original or mutant allele, which may be distinguishable by PCR or phenotypic analysis.

For transcriptome analysis in *Halobacterium *sp. NRC-1, both PCR microarray and Agilent *in situ *synthesized oligonucleotide [37] microarray platforms have been employed and used successfully for physiological and genetic studies [23-26]. For oligonucleotide arrays, probes of 60-nucleotide lengths were designed for 2474 ORFs utilizing the program OligoPicker [38]. Neither the relatively high average GC content of the chromosome nor the variations in GC content in different regions of *Halobacterium *sp. NRC-1 were problematic in the probe design and exceptionally high data quality was obtained, with low occurrence of data outliers due to non-uniform spot morphology, background noise, or spot-to-spot variation for replicate experiments.

The *Halobacterium *sp. NRC-1 proteome has also been extensively analyzed by liquid chromatography-tandem mass spectroscopy (LC/MS/MS) [39,40], and a total of 888 proteins were identified in whole cell lysates. The proteome from a similar *Halobacterium *species was analyzed by 2D-GE and MALDI-TOF mass spectrometric analysis of tryptic fragments and a reference map was established [41,42].

## Experimental studies of gene systems

Two types of gene systems have been studied in *Halobacterium *sp. NRC-1: those relevant to success in their extreme environment, such as the purple membrane, gas vesicles, DNA repair and anaerobic physiology, and those that are similar to fundamental eukaryotic processes, such as DNA replication, and transcription systems. Some studies were initiated prior to genome sequencing using genetic approaches have been advanced via genomic, bioinformatic, or functional genomic work, during the post-genomic period.

### DNA replication and repair

*Halobacterium *sp. NRC-1 provided the first opportunity for isolating autonomously replicating sequences from an archaeon and this was accomplished for both the chromosome and the large extrachromosomal replicon, pNRC100 [32,43]. The replicating sequences, putative *in vivo *origins of replication, were isolated and cloned using their ability to endow autonomous replication ability on *E. coli *plasmids containing a selectable mevinolin resistance (Mev^r^) marker. For the chromosome, a directed approach was taken to investigate whether chromosomal loci proximal to *orc/cdc6 *homologs possessed autonomous replication ability. A region of 2.3 kb containing the *orc7 *gene (one of ten eukaryotic-type origin recognition complex homologs), plus 750 bp upstream of the *orc7 *gene translational start, displayed replication ability [43]. A nearly perfect inverted repeat of 33 bp flanking an extremely AT-rich stretch of 189 bp (56% GC) was found in the upstream region. This *ori *region corresponded to one of two chromosomal inflection points in cumulative GC-skew analysis (which has been used for bioinformatic prediction of replication origins) [28,44]. The *ori *region was also conserved in the genome sequences of other halophilic archaea, including *Haloarcula marismortui *and *Haloferax volcanii *[43]. However, the region surrounding another *orc *gene near a second inflection point was not able to confer replication ability, suggesting the existence of only a single chromosomal origin in *Halobacterium *sp. NRC-1.

Previously, an autonomously replicating region of pNRC100, containing a 550 bp AT-rich region upstream of the *rep*H gene, was isolated using similar methodology [32]. However, in this case, the nature of the gene and the upstream region was unlike that found on any other replicon, except pHH, a closely related plasmid in another *Halobacterium *strain [45]. For the pNRC100 replication region, autonomous replication was disrupted by linker scanning mutagenesis in *rep*H, which was necessary for plasmid replication; however, the upstream region could be interrupted and partially deleted without knocking out replication ability. Genome sequencing showed that the *rep*H gene region is present in both pNRC100 and pNRC200, and therefore may be involved in the replication of both replicons. Interestingly, the latter replicon also contains multiple *orc *gene homologs, the functions of which are unknown [9-11]. In addition to providing a genetic approach to DNA replication studies in archaea, the availability of *Halobacterium *replicating sequences has facilitated the development of vectors, including both shuttle plasmids and expression vectors [19].

The number of replication origins and their coordination in *Halobacterium *sp. NRC-1 has been of significant interest. The euryarchaeon, *Pyrococcus abyssi*, was shown to contain a single chromosomal replication origin composed of similar large inverted repeats 5' to the only *orc/cdc6 *gene of this archaeon [46,47]. However, the crenarchaea, *Sulfolobus solfataricus *and *S. acidocaldarius*, were reported to contain two or three chromosomal replication origins proximal to multiple *orc/cdc6 *genes in their genomes [48,49]. In *Halobacterium *sp. NRC-1, a euryarchaeon, the roles and relationships of multiple (10) *orc/cdc6 *and *rep*H genes in replication require further experimentation for complete understanding.

Repair of DNA damage has been investigated in *Halobacterium *sp. NRC-1 because of the observed high levels of radiation resistance and the presence of homologs of both bacterial and eukaryotic-type repair genes [50]. For UV resistance, biochemical and genetic work demonstrated the presence of cyclobutane pyrimidine dimer photolyase activity, corresponding to one of two *phr *gene homologs in the genome [51]. Photolyase catalyzes the photoreversal of primary photoadducts from UV radiation, a process called photoreactivation, which is widely distributed in nature. In two DNA microarray studies, the transcriptional response of cells to UV irradiation was studied [24,26]; however, different results were obtained. In one study using 30–70 J/m^2^, specific induction of *rad*A1 and replication factor A genes (*rfa*3, *rfa*8, and *ral*) was observed [26]. In the other study, using substantially higher doses, a large number of unexplained changes resulted, probably caused by an overwhelming of the cellular repair systems and other physiological perturbations that were introduced [24]. In a biochemical study of the crenarchaeon, *Sulfolobus solfataricus*, replication factor A was found to bind to UV damaged DNA [52]. In a study of responses to high energy radiation (γ) and desiccation, both of which lead to extensive double-strand DNA breaks, resistance to these conditions was correlated, and protective effects of salts and membrane pigments were observed [53].

### The purple membrane regulon

The purple membrane of *Halobacterium *sp. NRC-1 contains the light-driven proton pump, bacteriorhodopsin, a complex of a protein, bacterio-opsin (*bop *gene product), and a retinal chromophore (Fig. 1). The purple membrane allows cells to grow phototrophically under high illumination, which may be important for survival under microaerobic and other stressful conditions [5,54]. A combination of genetic, bioinformatic, and transcriptional analysis showed the involvement of the *bop *gene cluster [23,54]. The cluster includes, in addition to *bop, crt*B1 and *brp*, coding the first and last committed steps of retinal synthesis, *blp*, a gene of unknown function, and *bat*, the sensor-activator gene. The Bat sequence predicted a complex protein consisting of a GAF (cGMP-binding) domain, PAS/PAC (redox-sensing) domain, and C-terminal DNA-binding helix-turn-helix (HTH) motif [54,55]. Additional Bat-like putative regulatory genes have been found in the genome, and together are likely to be responsible for the complex response of this archaeon to light and oxygen. The role of *brp *in the oxidative cleavage of β-carotene to form retinal was shown using the *ura*3-based gene knockout system, which also led to the identification of an unlinked gene, *blh*, capable of performing the same function [21]. Another unlinked gene involved in retinal biosynthesis, encoding lycopene cyclase (*crt*Y), was also identified by a genetic knockout [56].

The *bop *gene promoter region was studied by a combination of saturation mutagenesis and bioinformatic analysis [57-60]. The TATA-box sequence in the promoter deviates significantly from the archaeal consensus, suggesting the involvement of novel transcription factors in its recognition [58]. In this context, the finding of 6 Tbp (TATA-binding protein) genes and 7 Tfb (transcription factor B) genes in the *Halobacterium *sp. NRC-1 genome suggested the use of alternative Tbp-Tfb pairs in promoter selection and transcriptional regulation, similar to that proposed for higher organisms [60,61]. Mutagenesis also revealed a regulatory site, UAS, 5' to *bop *[59]. Additionally, UAS sites were found near two retinal synthesis genes, *brp *and *crt*B1, which are coordinately regulated with *bop *[54]. Similarities of the UAS and Bat regulator to diverse organisms, including a plant and a γ-proteobacterium, suggested an ancient origin for this regulon [11,54].

### Anaerobic metabolism

The metabolic capability of *Halobacterium *sp. NRC-1 includes anaerobic respiration using DMSO and TMAO as electron acceptors and fermentation with arginine via the arginine deiminase pathway (Fig. 1) [62,63]. The capability for anaerobic growth is likely advantageous for the organism in its natural habitat as high salt concentrations and elevated temperatures, together with high cell densities, reduce the availability of molecular oxygen. In a recent study of anaerobic respiration in *Halobacterium *sp. NRC-1 [25], bioinformatic and transcriptional analyses, and gene knockouts showed it to harbor a bifunctional DMSO/TMAO reductase that is encoded by the *dms*REABCD operon. This reductase is more closely related to NarG-type nitrate reductases than to bacterial DMSO/TMAO reductases, although phylogenetic analysis was inconclusive about its evolutionary origin. Whole-genome oligonucleotide microarray studies showed that the transcript level of the *dms *operon is strongly induced under anaerobic conditions [25]. Gene knockouts showed that expression of the *dms *operon is under positive transcriptional control of the regulator, DmsR. The C-terminal region of DmsR contains an HTH DNA-binding motif similar to that of the *bop *gene activator, Bat [19,25]. DNA microarray analysis also indicated that *Halobacterium *stays primed for aerobic respiration even under anaerobic conditions. This regulatory scheme could result in a fast metabolic response to oxygen as electron acceptor, when available. It is complemented by the increased abundance of gas vesicles (see below) under anaerobic conditions, allowing the cells to float to more aerobic zones in the water column. In a different study where *Halobacterium *strains overproducing or lacking purple membrane (generated by extensive chemical mutagenesis) were compared, it was deduced that during phototrophy, genes required for arginine fermentation are repressed [23].

### Gas vesicle biogenesis

Gas vesicles are hollow, buoyant protein structures that allow *Halobacterium *sp. NRC-1 to float, increasing their access to light and oxygen. A large gene cluster on pNRC100 (*gvp*MLKJIHGFEDACNO) is necessary and sufficient to produce gas vesicles [64-69]. Transcript mapping established the presence of divergent promoters in the *gvp*D-A intergenic region [66,67] and DNA microarrays confirmed that essentially all of the *gvp *genes are inducible under microaerobic, anaerobic, and other stressful conditions [25]. Rightward transcription of *gvp*A and *gvp*C, encoding the two most abundant gas vesicle proteins, was induced in early exponential growth phase [69]. Transcription was inhibited by aeration and by the addition of a DNA gyrase inhibitor, suggesting that increased DNA supercoiling is important for activation of *gvp*A transcription. A combination of genetic and bioinformatic analysis suggested that GvpE functions as an activator, with a leucine-zipper motif mediating dimer formation, and that GvpD, which contains an NTP-binding site, likely functions as a repressor by blocking GvpE-mediated activation [67,70].

The protein composition of *Halobacterium *sp. NRC-1 gas vesicles was studied using immunoblotting [71]. In addition to GvpA and GvpC, two small acidic polypeptides, GvpJ and GvpM, similar to GvpA were also identified as being present in the structure, and their function in determining vesicle membrane conformation was proposed [71]. The GvpC protein, which contains a motif that is repeated 7–8 times, and is predicted to be involved in binding to GvpA, was shown to be present on the surface of vesicles. Recombinant GvpC fusion proteins were found to bind to the surface of gas vesicles and are being used in a biotechnology application as an antigen delivery system [72,73]. Three additional proteins, GvpF, G, and L, were also observed in *Halobacterium *NRC-1 gas vesicles, and coiled-coil domains were identified in GvpF and GvpL, suggestive of self-association [71]. This was further confirmed by the observation of laddering of GvpL protein on gels. These genetic and biochemical results are consistent with the findings of comparative genomic studies, which showed that the corresponding *gvp *genes are present in all gas vesicle containing organisms examined [71,74-77].

### Arsenic resistance

*Halobacterium *sp. NRC-1 is resistant to arsenic, a heavy metal which is frequently found in hypersaline environments [22]. A gene cluster on pNRC100, *ars*MR*2*ADR*1*C, organized into three operons, was shown to be involved through construction and analysis of gene knockouts. Deletion of the *ars*ADRC gene region increased sensitivity to arsenite. However, knockout of a putative *ars*B gene homolog on the chromosome showed no phenotypic effect, suggesting the existence of a novel arsenite pump in *Halobacterium *sp. NRC-1. Interestingly, knockout of the *ars*M gene also produced increased sensitivity to arsenite, indicating a second novel mechanism of arsenic resistance involving an arsenite methyltransferase. The arsenite resistance elements were shown to be regulated, with resistance to arsenic being inducible by exposure to a sublethal concentration of the metal [22]. These results are consistent with *Halobacterium *sp. NRC-1 containing two arsenite detoxification systems.

### Coenzyme B_12 _metabolism

Coenzyme B_12 _metabolism has been explored in *Halobacterium *sp. NRC-1 using a combination of physiological and genetic approaches [78-81]. Salvaging of coenzyme B_12 _precursors from the environment was shown to require a previously unidentified amidohydrolase, the *cbi*Z gene product, which converts adenosylcobinamide to adenosylcobyric acid, an intermediate of the *de novo *coenzyme B_12 _biosynthetic route [80]. The salvaging of coenzyme B_12 _precursors by the CbiZ enzyme appears to be a uniquely archaeal strategy, because all of the genomes of B_12_-producing archaea have a *cbi*Z ortholog. Genetic studies showed that uptake of cobalamins for *Halobacterium *occurs via an ABC transporter similar to the bacterial Btu system [81].

### Other studies

Several additional experimental studies have been carried out on *Halobacterium *sp. NRC-1 genes, e.g. using *Escherichia coli *as host for complementation analysis and heterologous expression. An RNase H similar to the Type 1 bacterial enzyme, required for primer removal in DNA replication, was found to suppress the temperature-sensitive growth defect in *E. coli*, although the basic region present in the *E. coli *protein is absent in the haloarchaeal protein [82]. The haloarchaeal enzyme was also shown to cleave an Okazaki fragment-like substrate, consistent with its function in DNA replication. In another study, the molecular recognition of a tRNA_Cys _by the bacterial-type cysteinyl-tRNA synthetase of *Halobacterium *sp. NRC-1 was studied after expression in *E. coli *and showed similarities to its bacterial counterpart [83].

## Bioinformatic studies

A large number of bioinformatics investigations have been carried out on *Halobacterium *sp. NRC-1 since genome sequencing. They include studies of protein acidity, proteome structure prediction, and phylogenetic analysis of genes that may have been acquired through lateral gene transfer (LGT), as well as a number of broad or narrowly focused comparative studies.

### Acidic proteome

Isoelectric point analysis of the predicted proteome of *Halobacterium *sp. NRC-1 provided deep insight into the ability of this organism to survive under hypersaline conditions [9-11,28]. A dramatically low average pI (~4.9) was found *Halobacterium *proteins and the generality of this observation was confirmed through analysis of subsequent haloarchaeal genome sequences. In contrast, the median pI of nearly all non-halophilic proteomes has been found to be close to neutral, usually with a bimodal distribution of basic (pI ~10) and acidic (pI ~5) proteins. Notable exceptions were for some methanogenic archaea and an extremely halophilic bacterium, *Salinibacter ruber*, which sometimes coexist with *Halobacterium *in hypersaline environments and also contain relatively high internal salt concentrations [84-86]. The acidity of halophilic proteins likely helps maintain their function through increased solvation in an intracellular milieu with markedly reduced water activity [87].

**Figure 3:**
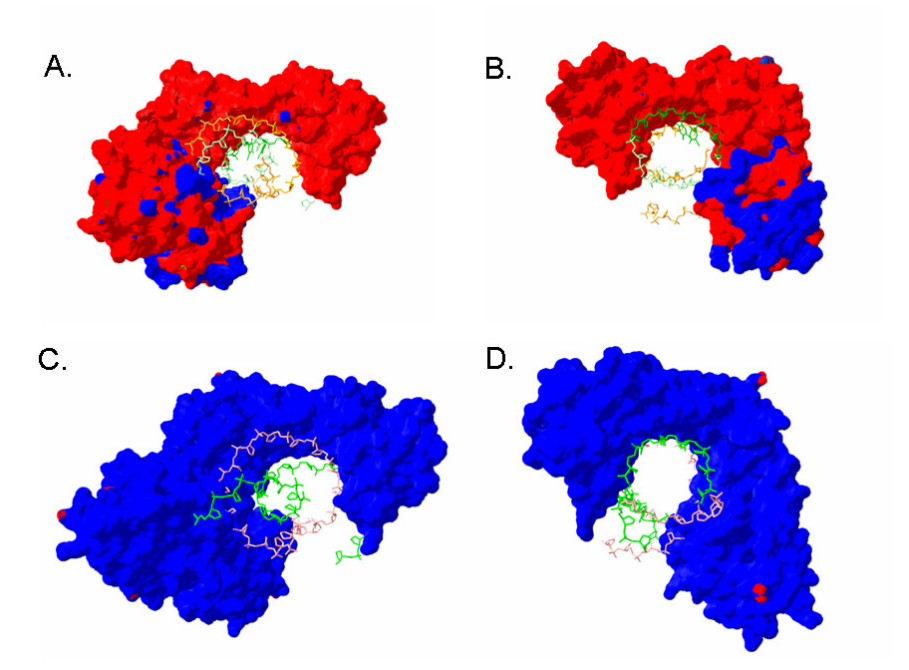
**Surface charge comparisons of the TBP-TFB-DNA complexes in *Halobacterium *and *Homo sapiens*. **Acidic character of proteins is indicated by red and basic by blue. DNA strands are green (coding) and pink or orange (non-coding). Panels A and B show the modeled complex in *Halobacterium *using a dielectric constant of 48.4 (NaCl concentration of 5 M) while panels C and D show the *Homo sapiens *complexes using a dielectric constant of 80.0 (NaCl concentration of 0 M). Panels A and C show transcription going into the plane while B and D show transcription coming out of the plane.

The acidic pI of *Halobacterium *proteins was correlated to a high concentration of surface negative charge in modeled structures [28]. A transcription factor (TbpE) and a topoisomerase subunit (GyrA) both showed significantly higher surface negative charges when compared to their homologs in non-halophilic organisms using a Coulomb charge calculation. Although there is an overall reduction in deprotonation of acidic residues on the protein surface at 5 M NaCl as the result of a decrease in the dielectric constant [88], the surface charges of most proteins were still highly negative. For example, when the Tbp-Tfb-DNA complex was modeled using a dielectric constant reflecting intracellular conditions, a dramatic difference in surface charges, compared to the human complex, was still observed (Fig. 3). This pattern of increased negative charge and lowered pI was observed for the vast majority of haloarchaeal proteins when compared to their homologs.

In another study, *de novo *structure prediction was conducted on 1,185 proteins and protein domains of *Halobacterium *sp. NRC-1 [89]. Putative functions were predicted by searching the Protein DataBank. Three examples, chemotaxis proteins, possible prophage polypeptides and archaeal transcriptional regulators, were highlighted in the published report.

### Evolution and lateral gene transfer

**Figure 4:**
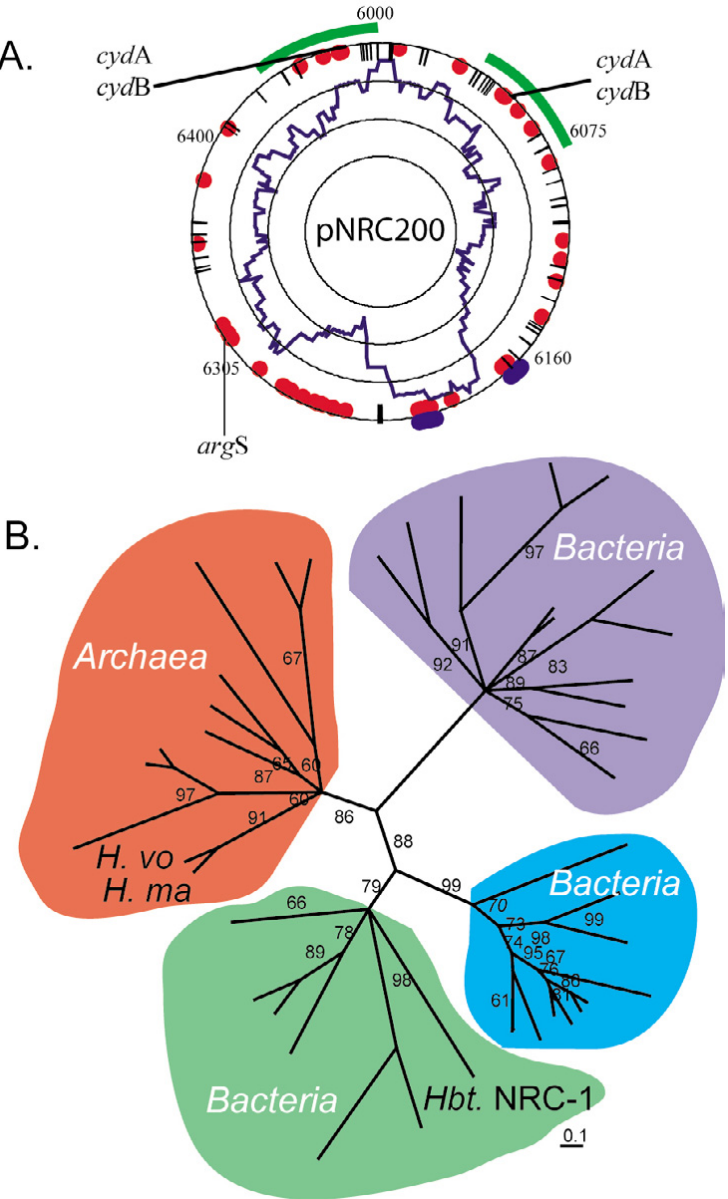
**"Bacterial" gene content in the *Halobacterium *sp. NRC-1 replicon pNRC200. **A. Average bacterial character is plotted in a 40 ORF window (innermost plot in blue). Individual genes closely related to bacteria in Blast analysis are shown with blue dots while those identified through COG analysis are shown as red dots. IS elements are indicated by lines on the outermost circle. B. Quartet puzzling maximum-likelihood phylogenetic tree of ArgS sequences from archaea and three classes of bacteria. A probable LGT of an *arg*S gene from a bacterium to *Halobacterium *(*Hbt*. NRC-1) is apparent. Two other haloarchaea (*Haloferax volcanii*: *H. vo*; *Haloarcula marismortui*: *H. ma*) contain archaeal-type ArgS-coding genes.

Initial whole-genome phylogenetic analysis of *Halobacterium *sp. NRC-1 confirmed the archaeal status of *Halobacterium *sp. NRC-1 [10], but also noted interesting similarities to the Gram-positive spore-forming bacterium, *Bacillus subtilis*, and the radiation-resistant bacterium, *Deinococcus radiodurans*. Subsequent whole-genome analysis using a larger number of completed genomes produced phylogenetic trees with *Halobacterium *branching near the base of the archaeal branch, or even within the bacteria [90,91]. This was in contrast to phylogenetic analysis based on concatenated sequences of transcription and transcriptional machinery, which produced results similar to 16S rRNA analysis placing *Halobacterium *within the archaeal clade [92]. One interpretation accounting for these incongruent results was that many LGTs have occurred between some bacteria and haloarchaea, although this conclusion appears to be open to some debate (Fig. 4) [93].

In spite of the initial uncertainties, several cases for LGT now appear to be convincing. Some components of the electron transport chain and biosynthetic proteins were found to have gene organization identical to homologous *E. coli *operons (*nuo *genes, coding subunits of NADH dehydrogenase; *cox *genes, coding subunits of cytochrome *c *oxidase; and *men *genes, coding for menaquinone biosynthesis) [28]. GC composition analysis showed these genes to deviate significantly from nearby *Halobacterium *genes and a phylogenetic study indicated their grouping with bacterial genes. A further detailed study of the *nuo*I gene showed clear phylogenetic affinity with proteobacteria [94]. These studies suggested that haloarchaea may have adapted to an oxidizing atmosphere by acquiring components of the electron transport chain through LGT events from bacteria [11].

A relatively recent case of LGT is the arginyl synthetase (*arg*S) gene of *Halobacterium *sp. NRC-1, which is found on pNRC200 [9]. Although its GC composition did not differ significantly from the genomic average, maximum-likelihood phylogenetic analysis showed that ArgS from *Halobacterium *does not group with other archaeal (including other haloarchaeal) ArgS proteins, but is most closely related to a class of ArgS proteins from bacteria (Fig. 4). Given the essential nature of arginyl synthetases to protein synthesis, it is likely that a bacterial *arg*S gene was captured and the archaeal *arg*S was subsequently lost in *Halobacterium *sp. NRC-1. Another example of orthologous gene displacement has been found in studies of lipid biosynthesis genes [95].

One of the most interesting evolutionary questions concerning *Halobacterium *centers on the origin of the light-driven proton pump, bacteriorhodopsin [11]. Such retinal chromoproteins (bacteriorhodopsin, halorhodopsin and sensory rhodopsins) originally discovered in haloarchaea have now been found in diverse bacteria and eukaryotes and therefore may have evolved before the divergence of the three domains of life [96]. Alternatively, occurrence in relatively few and diverse clades, e.g. oceanic planktonic bacteria, some fungi, and haloarchaea, suggests dispersal by LGT. Although phylogenetic analysis has thus far been inconclusive, spectroscopic characteristics have suggested the possible co-evolution of retinal with chlorophyll-based pigments [11,97].

### Other bioinformatics studies

An interesting study showed the importance of the Tat protein export pathway in *Halobacterium *sp. NRC-1 [98,99]. This pathway allows transmembrane transport of proteins in a fully folded conformation and is used for export of a moderate number of proteins in bacteria, e.g. those binding cofactors such as iron-sulfur clusters and molybdopterin. However, in *Halobacterium *sp. NRC-1, the majority of exported proteins are predicted to use the Tat pathway. This has been explained as an adaptation to the highly saline conditions, which may require intracellular folding for protein stability [98,99]. In another study, several poorly conserved open reading frames (ORFans) of *Halobacterium *sp. NRC-1, with apparent paralogs in the genome, but with no clear homologs in other organisms, were shown to be transcribed. The results indicated that all of the studied paralogous ORFans corresponded to real genes, including those comprising relatively short proteins [100]. Two comparative genomics studies have appeared, one comparing information transfer genes specifically among haloarchaeal species [101], and another comparing stress response genes in *Halobacterium *sp. NRC-1, *E. coli*, and *Drosophila melanogaster *[102].

## Future prospects

Haloarchaea represent excellent experimental models for extremophile biology and for fundamental aspects of archaeal and eukaryotic biology. They also serve as resources for theoretical questions of evolutionary biology and astrobiology [103]. For *Halobacterium *sp. NRC-1, the determination of the complete genome sequence and the development of many post-genomic experimental techniques, including gene knockout capability, DNA microarrays, and proteomics, as well as *in silico *bioinformatics approaches, have elevated this organism to the status of a leading model system among extremophiles and archaea. An advantage of post-genomic studies on *Halobacterium *sp. NRC-1 is that they may be conducted using well-characterized isogenic strains. As a result, future research efforts on this model system are likely to contribute significantly to broadening our understanding of fundamental biological concepts and ultimately testing our predictive powers.

## Competing interests

The author(s) declare that they have no competing interests.

## Authors' contributions

All authors participated in drafting of this manuscript.
